# Screening for diabetic retinopathy at a health centre in South Africa: A cross-sectional study

**DOI:** 10.4102/jphia.v16i1.681

**Published:** 2025-01-14

**Authors:** Ntokozo Zulu, Patrick Ngassa Piotie, Elizabeth M. Webb, Wezi G. Maphenduka, Steve Cook, Paul Rheeder

**Affiliations:** 1 School of Health Systems and Public Health, Faculty of Health Sciences, University of Pretoria, Pretoria, South Africa; 2 University of Pretoria Diabetes Research Centre, Faculty of Health Sciences, University of Pretoria, Pretoria, South Africa; 3 Department of Ophthalmology, Faculty of Health Sciences, University of Pretoria, Pretoria, South Africa; 4 The Eye Centre, East London, South Africa; 5 Department of Ophthalmology, Faculty of Medicine and Health Sciences, Walter Sisulu University, East London, South Africa; 6 Department of Internal Medicine, Faculty of Health Sciences, University of Pretoria, Pretoria, South Africa

**Keywords:** diabetic retinopathy, screening, primary care, diabetes, non-mydriatic photography, AI, microvascular complications, fundus camera

## Abstract

**Background:**

In South Africa, screening for diabetic retinopathy (DR) is non-existent at the primary healthcare (PHC) level because of the absence of a screening programme. This leads to preventable vision loss.

**Aim:**

To describe the clinical characteristics and outcomes of eye screenings and subsequent referrals.

**Setting:**

Laudium Community Health Centre (CHC), a PHC facility in Tshwane.

**Methods:**

We conducted a cross-sectional study from February 2022 to August 2022. Individuals with diabetes were screened for eye complications using visual acuity testing, intraocular pressure measurement, and fundoscopy with a non-mydriatic digital fundus camera. Fundus images were analysed by an optometrist and an artificial intelligence (AI) programme. Demographic and clinical data were collected.

**Results:**

A total of 120 participants were included, with the majority (60.7%) from Laudium CHC. Most participants (64.2%) were on oral agents, and 66.7% were women. The mean haemoglobin A1c (HbA1c) was 8.3%, with a median diabetes duration of 8 years. Artificial intelligence detected more glaucoma cases (17.5% vs 9.2%) and DR (23.3% vs 15.8%) compared to the optometrist. In contrast, the optometrist identified more cases of macula pathology (29.2% vs 19.2%). Participants (*n* = 79) were referred to an ophthalmologist for diagnosis confirmation and management.

**Conclusion:**

The study revealed that while DR was not highly prevalent among PHC patients with diabetes, there was a significant referral rate for other ocular complications. Artificial intelligence can enhance early detection and improve efficiency.

**Contribution:**

The findings underscore the need to integrate diabetes eye screening programmes into PHC services for people living with diabetes.

## Introduction

Diabetes affects approximately 537 million people worldwide, with Africa accounting for around 24 million.^1^ By 2045, this number is expected to double. In South Africa, an estimated 4.2 million individuals aged 20–79 years were living with diabetes in 2021, representing over 10.8% of the population.^1^

Diabetes significantly impacts healthcare systems because of the high prevalence and associated complications. Poor glucose control leads to macrovascular issues (such as peripheral arterial disease, stroke, and coronary artery disease) and microvascular complications (including retinopathy, neuropathy and nephropathy).

Over half of all blindness cases in Africa are preventable, with cataract being the leading cause.^2^ However, conditions like age-related macular degeneration (ARMD), glaucoma, and diabetic retinopathy (DR) are increasingly common. Diabetic retinopathy, the most common microvascular complication of diabetes, affects approximately one-third of people with diabetes globally.^3^ In 2020, DR was the fifth leading cause of blindness among individuals aged 50 years and older globally, accounting for 2.9 million cases of moderate to severe vision impairment and over 0.9 million cases of blindness.^4^ Diabetic retinopathy was the only cause of blindness that increased in age-standardised prevalence from 1990 to 2020.^4^

In South Africa, DR prevalence has been estimated in various hospital-based and primary-care-based studies. A systematic review reported a range from 7.6% to 62.4%.^5^ A 2014 study found a 39% prevalence at a tertiary diabetes clinic in Durban,^6^ while another reported a 24.9% prevalence at primary care clinics in the Tshwane district.^7^

The World Health Organization’s strategy for preventing avoidable blindness emphasises disease control, human resources development, and enhanced infrastructure and technology.^8^ Primary healthcare and community-based interventions are crucial for effectively controlling visual impairment.^9^

In high-income countries, DR screening programmes are well-structured and widely implemented. The United Kingdom’s (UKs) National Diabetic Eye Screening Programme invites individuals with diabetes aged 12 and older for annual digital retinal photography screenings.^10^ In the United States (US), screening is often covered by health insurance plans, including Medicare, although access and quality vary.^11^ Australia is integrating DR screening into primary care, but faces challenges like limited retinal cameras and low awareness among general practitioners.^12^

In low- and middle-income countries (LMICs), DR screening programmes face challenges such as limited resources, a lack of trained personnel, and insufficient infrastructure. Successful models include telemedicine and mobile screening units in countries like India and Botswana, which use non-mydriatic fundus cameras to reach rural areas.^13^ Brazil has increased coverage significantly, although access remains uneven across regions.^13^ Mexico and Costa Rica have made progress in developing national policies with varying regional implementation.^13^ In some instances, artificial intelligence (AI) is used to address challenges like a lack of trained personnel or limited resources by developing automated DR detection algorithms.^14,15^ By integrating AI into DR screening programmes, healthcare systems can leverage technology to enhance diagnostic accuracy, improve resource allocation, and ultimately provide better care for individuals with diabetes.^16^ Artificial intelligence technologies can be integrated into mobile screening units and telemedicine platforms, extending the reach of eye care services to remote and rural populations.

In South Africa, screening for DR is non-existent at the PHC level.^17,18^ Despite recommendations from the National Department of Health^19^ and professional organisations like the Ophthalmology Society of Southern Africa (OSSA)^20^ and the Society for Endocrinology, Metabolism and Diabetes of South Africa (SEMDSA),^21^ there is no national DR screening programme. As a result, many individuals with diabetes are not screened for DR and other comorbidities like glaucoma and ARMD.^20^ Society for Endocrinology, Metabolism and Diabetes of South Africa recommends annual retinal imaging using non-mydriatic fundus photography for DR screening.^21^ Barriers to implementation include budget constraints, competing healthcare priorities, and a lack of awareness about the importance of annual retinal exams.^22^

This study aimed to address the lack of DR screening at the PHC level by screening individuals with diabetes for eye complications at the Laudium Community Health Centre. Here, we describe the clinical characteristics and outcomes of these screenings and referrals.

## Research methods and design

### Study design

A cross-sectional study design was employed to assess the clinical outcomes of eye screening among individuals living with diabetes from February 2022 to August 2022.

### Setting

The study was conducted at the Laudium Community Health Centre (CHC), a PHC facility in the Tshwane district, Gauteng, South Africa. The centre operates 24 h a day and primarily serves the residents of Laudium and surrounding areas. It provides a range of services including chronic disease management, women’s health, child health, human immunodeficiency viruses (HIV) and acquired immunodeficiency syndrome (AIDS) care.

### Study population

Individuals with diabetes presenting for routine care at the Laudium CHC and those referred from nearby clinics including Olievenhoutbosch Ext 13 Clinic, Lyttelton Clinic, Rooihuiskraal Clinic, Eldoraigne Clinic, and Pierre Van Ryneveld Clinic were invited to participate in the study. All patients who arrived at the Laudium Eye Clinic during the study period, were 18 years or older, had confirmed type 1 or type 2 diabetes mellitus, were on diabetes medication, and provided informed consent were included.

### Sample size

A formal sample size calculation was not conducted for this study, as we used convenience sampling to include all eligible patients presenting at the eye clinic during the study period.

Out of the 125 participants seen by the optometrist over the study period, five were excluded because of inconsistent recording. The final analysis included 120 participants.

### Study procedures

Healthcare professionals from Laudium CHC and surrounding primary care clinics were informed about the availability of a fundus camera at the Laudium Eye Clinic for the duration of the study. They were invited to refer individuals living with diabetes for eye screening on a designated day each week. A researcher assisted the eye clinic staff in explaining the study’s procedures to referred patients and obtaining written informed consent. An optometrist conducted the eye examinations. Participants with referable eye conditions were referred to the Department of Ophthalmology at Kalafong Hospital, a tertiary academic hospital.

### Data collection

Data were collected using a questionnaire and datasheet. The following information was gathered:

- Demographic information: Age, gender, duration of diabetes, and medication usage.
- Clinical history: Medical history including diabetes-related complications and other chronic conditions.
- Laboratory investigations: HbA1c levels, lipid profiles, and creatinine levels were collected from patient files or from the National Health Laboratory Service online system.
- Ophthalmic examination: This included visual acuity testing using Snellen charts, intraocular pressure (IOP) measurement with an iCare^®^ tonometer, and fundoscopy. Fundus photography was performed using a Huzvit^®^ non-mydriatic digital fundus camera after pupil dilation with 1% Tropicamide as recommended by the ophthalmologist.

### Screening and grading

Each patient received a unique reference number, and their fundus images were stored electronically. The images were analysed by an optometrist and graded according to the Scottish Diabetic Retinopathy Grading Scheme,^23^ which includes:

- **R0:**   No diabetic retinopathy.
- **R1-R4:**   Ranges from mild background retinopathy to proliferative DR.
- **R6:**   Inadequate visualisation.
- **M0:**   No macular findings.
- **M1-M2:**   Observable and referrable maculopathy.

In addition to the optometrist’s assessment, the images were analysed by the AI-driven Singapore Eye LEsion Analyser (SELENA+), developed by EyRIS, to screen for diabetes-related eye diseases.^24^ Singapore Eye LEsion Analyser is designed to screen for DR, glaucoma and ARMD.

### Data analysis

Data were entered into EpiData 3.1 and analysed using STATA 17. Descriptive statistics were used to report frequencies, percentages, medians, means, and standard deviations (s.d.). We conducted an inter-rater reliability analysis to determine the degree of agreement between the optometrist’s assessment and the AI programme. The Cohen’s kappa score, which accounts for the possibility of agreement occurring by chance, was calculated.

### Ethical considerations

Ethical clearance to conduct this study was obtained from the University of Pretoria Faculty of Health Sciences Research Ethics Committee (No. 326/2022). In addition, permission to conduct the study at Laudium CHC was requested from the Tshwane Research Committee (National Health Research Database [NHRD] Reference Number: GP_202105_043).

All procedures performed involving human participants were in accordance with the ethical standards of the institutional research committee and with the 1964 Helsinki Declaration and its later amendments or comparable ethical standards. Written informed consent was obtained from all individual participants involved in the study. To maintain the confidentiality of data, all participant information was anonymised and assigned unique identification codes. Data were securely stored, and access was restricted to the research team.

## Results

The study included 120 participants from the Laudium CHC (*n* = 71, 60.7%), nearby PHC clinics (*n* = 14, 12.0%), and down-referrals from Kalafong Hospital (*n* = 32, 27.4%). Three participants’ referral sources were unknown.

The participants’ demographic characteristics and clinical history are summarised in Table 1. Of the participants, 66.7% were women. The median duration of diabetes was 8 years. Most participants (64.2%) were on oral medications, and 33.3% were using insulin. A history of other complications included previous heart disease (3.3%), strokes (2.5%), amputations (0.8%), and neuropathy (57.5%). Most participants (20%) reported a history of eye problems.

**TABLE 1:** Demographics and clinical history of participants seen at the Laudium Community Health Centre Eye Clinic for eye screening (*N* = 120).

Participant characteristics	*n*	%	Mean	s.d.	Median	IQR
Age (years)	-	-	60.0	11.9	-	-
Duration of diabetes (years)	-	-	-	-	8	4–15
**Gender**					
Women	80	66.7	-	-	-	-
Men	40	33.3	-	-	-	-
**Medication**			-	-	-	-
Orals only	77	64.2	-	-	-	-
Orals and insulin	39	32.5	-	-	-	-
History of eye disease	24	20.0	-	-	-	-
History of heart disease	4	3.3	-	-	-	-
History of stroke	3	2.5	-	-	-	-
History of amputation	1	0.8	-	-	-	-
History of peripheral neuropathy	69	57.5	-	-	-	-

s.d., standard deviation; IQR, interquartile range.

### Laboratory investigations

The mean haemoglobin A1c (HbA1c) was 8.3% among 78 participants, indicating suboptimal blood glucose control (Table 2). Low density lipoprotein (LDL) cholesterol (57 participants) and total cholesterol (61 participants) averaged 2.8 mmol/L (s.d.: 1.0) and 4.6 mmol/L (s.d.: 1.1), respectively.

**TABLE 2:** Clinical laboratory tests of participants seen at the Laudium Community Health Centre Eye Clinic (*N* = 120) for eye screening.

Diabetes control parameters	Tests available		Mean	s.d.
	*n*	%		
HbA1c (%)	78	65.0	8.3	2.2
Creatinine (µmol/L)	53	44.2	90.8	85.6
LDL cholesterol (mmol/L)	57	47.5	2.8	1.0
Total cholesterol (mmol/L)	61	50.8	4.6	1.1

HbA1c, haemoglobin A1c; LDL, low density lipoprotein; s.d., standard deviation.

### Visual acuity

Among the 120 participants, 70% had normal vision, while 18.3% were visually impaired, and 5.8% were severely impaired (Table 3).

**TABLE 3:** Visual acuity and intraocular pressure measures of participants seen at the Laudium Community Health Centre Eye Clinic (*N* = 120) using Snellen charts.

Visual acuity	*n*	%
**Normal vision**	84	70.0
6/6	25	20.8
6/7.5	16	13.3
6/9	13	10.8
6/12	30	25.0
**Visually impaired**	22	18.3
6/18	8	6.7
6/20	6	5.0
6/30	8	6.7
**Severely impaired**	7	5.8
6/60	7	5.8
No data	7	5.8
**Intraocular pressure (IOP)**	-	-
Normal IOP (10 mmHg – 20 mmHg)	103	85.8
Elevated IOP (>21 mmHg)	12	10.0
No data	5	4.2

CHC, Community Health Centre; IOP, intraocular pressure.

### Intraocular pressure

Intraocular pressure between 10 mmHg and 20 mmHg is considered normal. Values above 20 mmHg are deemed elevated and increase the risk of glaucoma. In our study, 12 participants (10.0%) had elevated IOP.

### Fundoscopy

Table 4 compares eye pathology diagnoses made by the optometrist and SELENA+ for 120 participants. The AI programme detected more glaucoma cases (17.5% vs 9.2%) and DR (23.3% vs 15.8%) compared to the optometrist. In contrast, the optometrist identified more cases of macula pathology (29.2% vs 19.2%). Both methods found a substantial number of participants with no pathology.

**TABLE 4:** Comparison of eye pathology diagnoses by optometrist and SELENA+ during screening at the Laudium Community Health Centre Eye Clinic.

Eye pathology	Optometrist		AI	
	*n*	%	*n*	%
Cataract	14	11.7	13	10.8
Glaucoma	11	9.2	21	17.5
Macula pathology	35	29.2	23	19.2
Diabetic retinopathy	19	15.8	28	23.3
No pathology	41	34.2	34	28.3
Not gradable	0	0.0	1	0.8

CHC, Community Health Centre; AI, artificial intelligence.

The inter-rater reliability analysis between the optometrist and the AI programme showed substantial agreement overall, with a Cohen’s kappa score of 0.787. Specifically, the AI demonstrated almost perfect agreement for cataract and no pathology, substantial agreement for DR and macula pathology, and moderate agreement for glaucoma, despite detecting more cases of DR than the optometrist.

### Participant referral and management

Following the optometrist’s assessment, 79 participants were referred to an ophthalmologist for confirmation of the diagnosis and further management. At the end of the study, referral outcomes were available for 25 participants (31.6%). Among these, seven were diagnosed with cataracts, 11 with glaucoma, and seven with DR.

Of the seven participants with cataracts, six underwent surgery, while one declined surgical treatment due to fear of post-surgical blindness. Among the 11 participants with glaucoma, two received immediate treatment for severe glaucoma, and the remaining nine were scheduled for follow-up appointments. Of the seven participants with DR, three had non-proliferative DR and were recommended for follow-up, two received Avastin intravitreal injections, and one underwent pan-retinal photocoagulation therapy.

## Discussion

This study highlights the critical need for integrating regular eye screenings into PHC for people living with diabetes. Our findings further demonstrate the potential benefits of using AI alongside traditional screening methods to improve detection and management of DR.

We found that glucose control among participants was acceptable, with a mean HbA1c of 8.3%. Previous studies reported suboptimal glucose levels in the Tshwane district, indicating a broader issue of inadequate glycaemic management in the region.^25,26,27^ Poor glucose control is a significant risk factor for the development and progression of DR.^6,7,28,29^ This finding underscores the importance of comprehensive diabetes management, which includes both regular eye screenings and maintaining effective glycaemic control.

In our study, 24.2% of the participants were visually impaired to severely impaired, which is similar to a study in which 21.5% of participants were reported to have visual impairment.^7^ These findings highlight the significant burden of vision impairment in people with diabetes.

The prevalence of DR in our study (15.3%) was relatively low compared to other studies conducted in South Africa, which have reported DR prevalence rates ranging from 25% to 63%.^6,7,30,31^ This discrepancy may be due to differences in study populations, screening methods, and healthcare access across different regions.

In a study reported by Mash et al.,^30^ despite the average duration of diabetes being only 7.4 years – similar to the average duration in our study – the rates of ocular complications were high. This suggests that even with a relatively short duration of diabetes, the risk of developing serious ocular complications remains significant. In our context, this could be explained by a failure to intensify treatment, leading to poor glycaemic control and subsequently higher rates of complications, including DR.^25^ Aggressive management of blood glucose levels is necessary to mitigate the risk of ocular complications in individuals with diabetes.

Integrating AI in screening for DR and other ocular pathologies has significant implications for clinical practice, especially in resource-limited settings. This study demonstrates the potential of AI to augment traditional screening methods and improve detection rates for various eye conditions. The AI programme in this study identified a higher prevalence of DR (23.3%) compared to the optometrist’s assessment (15.3%), suggesting that AI can potentially detect subtle or early signs of DR that might be missed by human examiners.^32,33^ Enhanced detection rates can lead to earlier intervention and better patient outcomes.

Even though the AI programme detected more cases of DR than the optometrist, the Cohen’s kappa score still indicated substantial agreement. This suggests that while the AI might be more sensitive in detecting DR, it generally agrees with the optometrist’s diagnoses. This synergy between AI and human expertise can enhance overall diagnostic accuracy and improve patient care outcomes.

### Study strengths and limitations

Conducting the study at Laudium CHC, a real-world primary care setting, enhanced the relevance and applicability of the findings to similar environments, particularly in resource-limited areas. However, we faced two significant challenges: (1) the limited number of patients that could be booked per week, and (2) the optometrist’s concerns about increased workload, which reduced the sample size and may have limited the generalisability of the findings to larger populations.

The study’s cross-sectional design provides only a snapshot in time, making it difficult to assess long-term outcomes and trends in DR progression and management. The comparison of diagnoses made by optometrists with those from the AI programme offers valuable insights into the potential for AI to complement and improve human clinical judgement, highlighting the importance of integrating AI into routine screening programmes to alleviate the burden on healthcare professionals and improve efficiency.^34^

Future research should focus on post-screening outcomes for DR, specifically the establishment and effectiveness of referral pathways. Understanding and improving the referral process is crucial to ensure patients receive appropriate follow-up care and treatment. Studies should investigate barriers and facilitators to successful referrals and the impact of timely interventions on patient outcomes.

## Conclusion

Routine screening for ocular pathologies in people living with diabetes is often missing in primary diabetes care in South Africa. This study underscores the significant benefits of incorporating retinal screening into PHC. Despite the relatively low prevalence of DR, the high referral rates for other ocular complications such as glaucoma, cataracts, and macula-related issues highlight the urgent need for regular eye examinations. The use of AI in screening programmes can further enhance early detection and improve efficiency. Translating existing guidelines into practical, widespread screening programmes, supported by AI technology, within PHC settings is essential to ensure timely intervention and prevent vision loss.
